# Evaluating Chondroitin Sulfate and Dermatan Sulfate Expression in Glial Scar to Determine Appropriate Intervention Time in Rats

**DOI:** 10.32598/bcn.9.10.405

**Published:** 2020-01-01

**Authors:** Sara Rezaei, Salar Bakhtiyari, Khairolah Assadollahi, Somayeh Heidarizadi, Ardashir Moayeri, Monireh Azizi

**Affiliations:** 1. Department of Anatomy, Faculty of Medicine, Ilam University of Medical Sciences, Ilam, Iran.; 2. Department of Biochemistry, Faculty of Medicine, Ilam University of Medical Sciences, Ilam, Iran.; 3. Department of Epidemiology, Faculty of Health, Ilam University of Medical Sciences, Ilam, Iran.; 4. Biotechnology and Medicinal Plants Research Center, Ilam University of Medical Sciences, Ilam, Iran.

**Keywords:** Spinal cord injury, Glial scar, Chondroitin sulfate, Dermatan sulfate

## Abstract

**Introduction:**

The proteoglycans of the extracellular matrix increases in the glial scar during spinal cord injury and significantly affects the inhibition of axonal regeneration.

**Methods:**

The results of injury therapies are limited due to the lack of identifying a timely therapeutic intervention. The present study aimed to investigate the glial scar Chondroitin Sulfate (CS) and Dermatan Sulfate (DS) levels at different post-injury times to determine the appropriate time for therapeutic intervention.

**Results:**

By this experimental study, 72 Wistar rats were randomly divided into 12 groups, as follows: control, sham, injured animals at 1, 2, 4, and 8 days, as well as 2, 4, 8, 12, 16, and 20 weeks post-injury. The animals in the injured groups were contused in the T10 segment of the spinal cord. The motor function of animals was assessed using the Basso, Beattie, and Bresnahan (BBB) test. Besides, the histological assessment was performed using Luxol Fast Blue and Bielshovisky Staining. The CS and DS levels of lesions were measured using the Enzyme-Linked Immunosorbent Assay (ELISA) method.

**Conclusion:**

The motor function assessment indicated a relative recovery over time. Histological results confirmed some regeneration in the injury site at 20 weeks post-injury. The ELISA results demonstrated a much higher level of DS than that of CS in the glial scar. Considering high levels of DS, compared to CS in the glial scar and its reduction from second weeks after SCI onwards, the second week after SCI seems to be the best time for therapeutic interventions in terms of scar permeability.

## Highlights

- The motor (BBB) test results indicated relative recovery after spinal cord injury by passing the time.
- The Luxol Fast Blue (LFB) staining results at 20 weeks post injury confirmed some meyelination in the injured area.
- Some axonal regeneration confirmed at 20 weeks post injury by Bielshovisky staining.
- In the spinal cord injured site Dermatan Sulfate (DS) amounts was much higher than Cratan Sulfate(CS).
- It seems that second week after SCI to be the best time for therapeutic approaches in terms of scar permeability based on ELISA results.

## Plain Language Summary

12 groups, including control, sham, injured animals were evaluated. The animals in the injured groups were contused in the T10 segment of the spinal cord. The motor function of animals was assessed using the Basso, Beattie, and Bresnahan (BBB) test. The CS and DS levels of lesions were measured using the Enzyme-Linked Immunosorbent Assay (ELISA) method. The achieved results revealed that the scar’s DS level was much higher than that of CS, and their levels reached to the maximum rate on the first day after injury. The motor function assessment indicated a relative recovery over time. Histological results confirmed some regeneration in the injury site at 20 weeks post-injury. Considering high levels of DS, compared to CS in the glial scar and its reduction from second weeks after SCI onwards, the second week after SCI seems to be the best time for therapeutic interventions in terms of scar permeability.

## 1. Introduction

Spinal Cord Injury (SCI) is among the most severe disabilities that significantly impact one’s life and causes extensive restrictions in life (Devivo, 2003). After severe SCI, the Central Nervous System (CNS) astrocytes form the main component of the glial scar by being transformed into reactive astrocytes, i.e., considered as large barriers to axonal regeneration (Matsui and Oohira, 2004; Rhodes & Fawcett, 2004). The rapid proliferation of astrocytes around the lesion is one of the special characteristics of the injury in all mammals (Fawcett and Asher, 1999).

After the injury, the resulting scar is composed of laminin growth-promoting molecules and cell adhesion molecules, growth factors, and some inhibitory molecules; however, axonal growth is inhibited due to the dominance of the inhibitory molecules at the injured site (Shields, Zhang, Burke, Gray, & Shields, 2008). Chondroitin Sulfate Proteoglycans (CSPGs) act as guidance and signaling molecules during the development and maintain the structural integrity of the intact CNS in specialized areas, such as the basement membrane, perineuronal nets, and the nods of Ranvier (Lau, Cua, Keough, Haylock-Jacobs, & Yong, 2013).

Studies suggested that the glial scar plays a key role in protecting intact tissues by surrounding the injured site (Faulkner et al., 2004; Myer, Gurkoff, Lee, Hovda, & Sofroniew, 2006; Rolls, Shechter, & Schwartz, 2009; Sofroniew, 2009), preventing inflammation and further degeneration of myelin; however, it inhibits axonal regeneration (Okada et al., 2006). Very limited regeneration ability of CNS neurons is mainly due to the release of inhibitory molecules in the CNS (Wilson, Forgione, & Fehlings, 2013). CSPG secreted by the reactive astrocytes forms the main components that inhibit the axons’ growth (Matsui and Oohira, 2004).

The CSPG level is upregulated and is considered as a strong inhibitor of axonal regeneration after traumatic injuries (Lau et al., 2013; Silver and Miller, 2004). Increased CSPG expression level after SCI has been reported by previous studies (Pasterkamp, Anderson, & Verhaagen, 200; Plant, Bates, & Bunge, 2001). Increased CSPG expression level at the injury site as well as at the sites far from the SCI contusion model was reported by Andrews et al. (2012). Researchers also argued that CSPG directly inhibits oligodendrocytes and progenitor cells’ growth process and their differentiation into mature oligodendrocytes in an in vitro model (Siebert & Osterhout, 2011). Therefore, considering the scar inhibitory effect on the axonal regeneration, the regeneration process, as well as reduced scar extent and destructed degeneration molecules, should be conducted as quickly as possible. However, it is important that the inhibition of factors causing scar formation to be avoided due to their primary protecting role (Ueno & Yamashita, 2008).

Problems, like incomplete transection and knife-cut, damage the spinal cord meninges and allow the peripheral cells to invade the injury site. These injuries induce changes, such as the upregulation of neurocan, brevican, and NG2 in CSPGs expression levels in the injured site (Tang, Davies, & Davies, 2003; Massey et al., 2008; Jones, Margolis, & Tuszynski, 2003); these are responsible for creating a chemical barrier to extend axons in the injury site (Fitch & Silver, 2008). However, little is known about changes in the CSPGs expression levels contusion SCI which almost accounts for half of all cases of clinical injuries (Norenberg, Smith, & Marcillo, 2004). Nevertheless, many researchers have achieved no significant recovery in this regard due to a lack of timely therapeutic interventions. Therefore, the current study aimed to determine the CS-DC levels in the glial scar at different times post SCI to determine the effective time of therapeutic intervention and reduce the inhibitory effect of scars.

## 2. Methods

A total of 72 male Wistar rats weighing 210±10 g were explored in this study. One week before the experiment onset and during the whole study period, the study animals were kept at Animals Care Center of the University under standard laboratory conditions, including easy access to water and food, 1212-h light-dark cycle, and a temperature of 22°C. The study animals were randomly divided into 12 groups (n=6) of control, sham, injured animals at 1, 2, 4, and 8 days, as well as 2, 4, 8, 12, 16, and 20 weeks post-injury. No action was taken in the control group, and laminectomy was the only action performed in the sham group.

All spinal cord-injured groups underwent laminectomy; then, contusion SCI was created in the T-10 segment of the spinal cord by dropping a 10gr weight from a height of 25mm (Wang et al., 2011). Moreover, the injured animals were kept in disinfected cages during the study period. They also received cefazolin [1mg/kg, Intraperitoneal (IP)], and saline (2–5 ml) for 5 days after injury. To assess the motor function, the Basso, Bresnahan, and Beattie (BBB) test with a scoring range of 0–21 was used (Basso, Beattie, & Bresnahan, 1996). A blind analysis was performed on the locomotor capacity of all groups by two individuals, and the final score was reported as the mean value given by these individuals.

The BBB test was conducted daily in the injured animals at 1, 2, and 4 days post-injury; in other groups, it was conducted after the first two days, then weekly until the end of the survey. At the end of the study period, the tissue samples of the injury sites were collected from three rats from each of the groups for histological studies. For this purpose, animals transcardially underwent initial perfusion fixation with normal saline followed by 10% normal saline after being anesthetized using ketamine/xylazine (60/6 mg/Kg). Then, after removing the injury site, the collected samples were placed in the same fixatives to undergo the secondary fixation for 24 hours. The LFB and Bielshovisky staining methods were used for the histological evaluation of tissue samples obtained from the injury site. Perfusion-fixed spinal cord tissue underwent the paraffin molding process after the passaging step; finally, 5μm thick slices were prepared.

The Luxol Fast Blue (LFB) and Bielshovisky staining methods were used respectively to assess the myelin level and the presence of nerve fibers in the injury site. For the injured spinal cord preparation for the Enzyme-Linked Immunosorbent Assay (ELISA) technique, the spinal cord was removed after anesthesia with ketamine/xylazine (60/6 mg/kg) and the reopening of the laminectomy area. Then, equal rostral and caudal cuts were prepared from all samples at a distance of 1 mm apart from the injury site center. The obtained samples were then washed with normal saline and maintained in a formaldehyde solution at −80°C. At the end of the study period (20^th^ week), all samples were transferred from the freezer to room temperature, and the protein extraction was performed.

The total protein of spinal cord tissue was extracted using the protein extraction solution (Cat. No. 17081, Bulldog Bi Company). Briefly, 10–20 mg tissue samples were prepared from the injury sites, and tissue homogenization was performed in 600 μl of protein extraction solution with protease inhibitor cocktail. Cells lysis was then continued by incubating the cells in the −20°C (freezer) for 20–30 min. The samples were centrifuged at 13000 rpm (4°C) for 5 minutes and the supernatant was transferred to 1.5 ml tube. Eventually, the protein concentration was determined using the Bradford’s method (Bradford, 1976). The levels of DS and CS were measured using commercially available kits according to their protocols (Rat Dermatan sulfate ELISA Kit: cat No. MBS263602-MyBioSour, USA, and Chondroitin sulfate Kit: SEB141Ra96, Designed by Cloud-Clone Crop, Uscn Life Science Inc. tests, USA). Finally, the levels of DS and CS normalized to the levels of total protein.

The statistical analysis was performed using the Minitab. The collected data were reported as Mean±SD at a significance level of P≤0.05. Inter-group comparisons were made using one-way Analysis of Variance (ANOVA) at a significance level of P≤0.05.

## 3. Results

**Figure 1.**
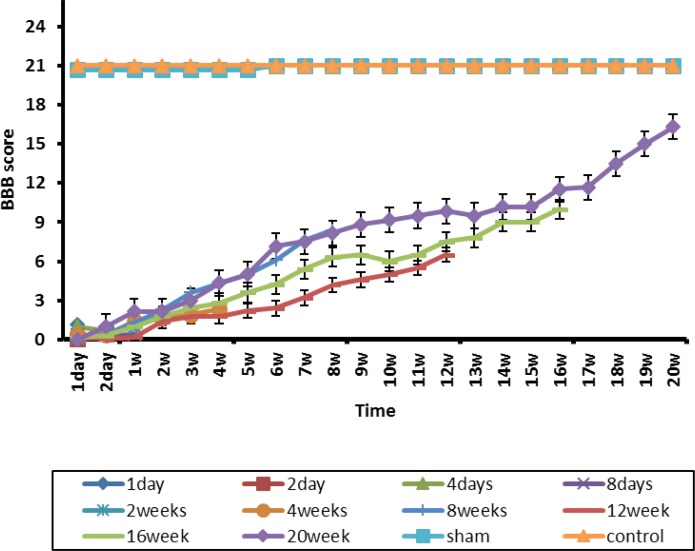
The BBB motor test in various groups from the beginning to the end of the 20^th^ week of study Results are expressed as Mean±SD, and the number of animals in each group were 6. A significant difference was observed between the control and sham groups, and the injured groups during the study period. The motor’s score of injured animals reached 16 at the end of the study (week 20).

Comparing the BBB test results indicated a significant difference between the control and sham groups, and the injured groups during the study period (P<0.05). However, there was no significant difference between the control and sham groups from beginning to the end of the study (week 20). Accordingly, the laminectomy alone does not impair the motor function in these animals, and all animals gained score 21 from the beginning to the end of the study (Figure 1).

A functional motor recovery with a slow pace was observed in the groups with spinal injury at the end of the second to the fifth weeks of the study. The recovery level was increased more quickly after the fifth week until the end of the study (week 20). This raise was to the extent that the motor score of injured animals reached to score 16 at the end of the study (Figure 1).

**Figure 2.**
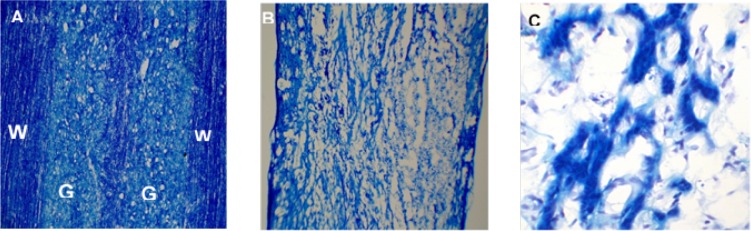
The LFB staining of spinal cord sections at level T10 segment A: Intact spinal cord in the sham group with the normal integration of white and gray matters; B and C: Injured spinal cord without any treatment intervention in 20 weeks after SCI lesion; an injured area in the 20-weeks post-injury was filled with some tissue and myelinated axons, which probably is a reason for some regeneration observed in these animals. The magnifications of images A, B, and C are 10×, 4×, and 40× respectively. W and G indicate the white and gray matters of the spinal cord, respectively.

To assess the myelin level, LFB staining was applied. The LFB staining results suggested the intactness of the spinal cord and the safety of laminectomy in the sham group (Figure 2A). The staining results in injured animals, at 20 weeks post-injury, also confirmed the filled cystic cavity and the presence of some myelination in the injured area (Figure 2B & C).

**Figure 3.**
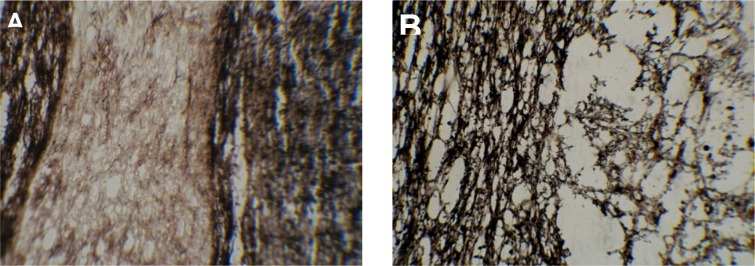
Bielschowsky staining of intact sham (A) and injured-T10 segment of spinal cord 20 weeks post-injury (B) A. The intact spinal cord was observed in the sham group; B. A cavity with some axon growth was observed in the spinal cord section 20 weeks after lesion. The axons’ existence in the cavity reflects some degree of regeneration. Magnification of image A is 10×, and image B is 40×.

To assess the presence of axons in the lesion, Bielshovisky staining was used. The results of this staining approach also highlighted the intactness of the spinal cord tissue and the absence of laminectomy injury in the sham group (Figure 3A). Some of the axons were also observed in injured animals at week 20 post-injury (Figure 3B).

**Figure 4.**
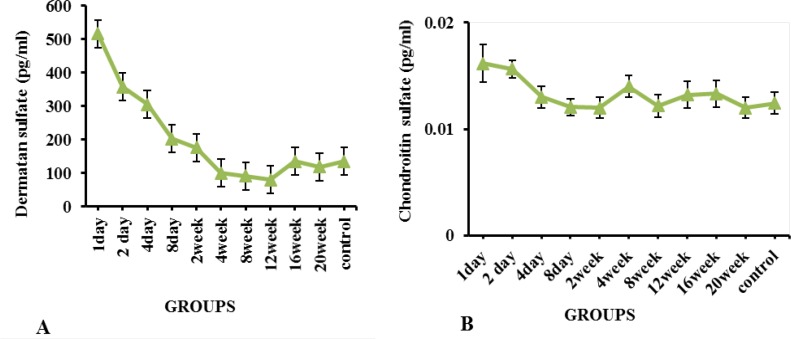
A. DS; and B: CS present levels at the injury site based on the ELISA technique results in all study groups The number of assessed animals in each group was 3, and the relevant results are expressed as Mean±SD. The DS levels were much higher than the CS levels in the scar all the time. The DS level was increased on the first and second days, and the decreasing trend was later observed to its minimum level in the 12^th^ week. The maximum CS level in the injury site was detected in the second-day post-injury, then decreased with a fluctuating trend and reached the same level as the control group in the 20^th^ week.

Glial scar consists of different ECM components, the expression levels of which vary at different times after the injury. In this study, the DC and CS levels at the injured site were evaluated using the ELISA technique at 24 hours to 20 weeks after the injury. The relevant results suggested that the DS was much higher than the CS in the scar at the times mentioned above (Figure 4). Furthermore, the DS level was increased on the first and second days, and the decreasing trend was later observed to the extent that the minimum level in the 12^th^ week. The DS level was equal and less than that of the control group in the 16^th^ and 20^th^ weeks, respectively (Figure 4A). The maximum CS level was observed in the injury site in the second-day post-injury; it was decreased with a fluctuating trend and reached to the same level as the normal control group in the 20^th^ week (Figure 4B).

## 4. Discussion

In this 20-week study, a relative recovery in the motor function was observed over time; the motor score of 16 was obtained for spinal cord-injured animals at the end of the study (week 20). The histological results supported the axonal regeneration and remyelination, though limited, in the injury site.

In a study on a photochemical model of spinal cord injury, Verdu et al. (2003) reported a BBB score of 18 for injured animals at the end of the 12^th^ week. Garcia-Alias, Lopez-Vales, Fores, Navarro, & Verdu. (2004) also obtained a BBB score of 15 for the acute photochemical SCI model in injured rats at the end of the 12^th^ week. However, in our study, the BBB scores of 6.5 and 16 were obtained at the end of the 12^th^ week and 20^th^ week, respectively. This data discrepancy may be due to differences in the severity of SCI; the severity of the photochemical SCI used in the study by Verdu and Garcia-Alias was milder than the contusion injury created in the present study. Fouad et al. (2005) also reported a score of 2.1 as the average motor score in injured adult rats with the complete severing of the spinal cord in the T7–T9 region. Lower motor recovery rate reported by Fouad, compared with the present study, is probably due to the extent and severity of injury created in Fouad’s study.

Richter et al. (2005) attributed post-injury limited regeneration to weak internal regeneration and the angiogenesis capacity of neurons. Mothe and Tator (2005) stated that the number of cells stimulated in response to the injury differed depending on the injury model; where stem/progenitor cells responses were locally distributed in the spinal cord transection model and systematically throughout the rostral and caudal regions of the spinal cord in the contusion/compression model. This broader response is probably because a wider region of spinal cord tissues was affected in the two injury models mentioned above. Additionally, the injury is somehow recovered when some of the stem/progenitor cells in the ependyma are recruited to the injury site, and the proliferation power is increased (Widenfalk, Lundstromer, Jubran, Brene, & Olson 2001). However, cell proliferation does not always lead to the restoration of the spinal cord function and sometimes fills only the created cavities (Ropper, 2001).

Various forms of plasticity have been reported in neurons of the cortex, brainstem, and spinal cord; to some extent, they could play a role in increasing post-injury compensation recovery (Weidner, Ner, Salimi, & Tuszynski, 2001). However, Raineteau et al. (2002) reported the reorganization of descending motor tracts in rats with SCI. The current study revealed an improved motor function, axonal regeneration, and remyelination without any therapeutic intervention that may be associated with the presence of stem/progenitor cells in the injured area, weak regeneration by the neurons at the injured site, angiogenesis, the reorganization of intact circuits, and even plasticity changes which has also been reported by Widenfalk et al. (2001).In the present study, the CS and DS levels in the glial scar, i.e., the main axonal regeneration barrier, were measured using the ELISA method at 24 hours to 20 weeks after the injury.

The achieved results revealed that the scar’s DS level was much higher than that of CS, and their levels reached to the maximum rate on the first day after injury. As a result, these proteoglycans play the main role in preventing axonal regeneration during the acute phase of glial scar formation. The glial scar contains different extracellular matrix components, which have various expressions’ levels at different times. A study explored the expression level of CSPGs (NG2, neurocan, the phosphagen, brevican, and versican V2) and tenascin-c during spinal cord scar, the transformation from the acute to the chronic phase within 24 hours to 6 months after injury.

Consequently, Tang et al. (2003) reported that the axonal growth was inhibited by these macromolecules. These researchers found a sharp increase in the level of neurocan, tenascin-C, and NG2 within 24 hours. Moreover, they stated that these molecules play a preventing role in the axonal regeneration in the glial scar formation during its acute phase. In Tang’s study, the maximum level of phosphacan brevican was observed one month after the injury. In contrast, the versican V2 level was reduced sharply, even in comparison with its levels in the intact tissue during the study period. Six months after the injury, neurocan, brevican, and NG2 levels in chronic scar tissue remained significantly higher than the control groups. Therefore, these CSPGs and tenascin-C in the extracellular matrix could be considered as important inhibitors of axonal regeneration during acute to chronic maturation of spinal cord scar tissue (Tang et al., 2003).

In the present study, using the ELISA method, the highest levels of CS and DS were observed within 24 and 48 hours after the injury, respectively. These changes led to the inhibition of axonal regeneration in the acute phase of injury. In contrast to Tang’s study, our results suggested that the CS level was sharply reduced after 48 hours, and following a fluctuation trend, its level reached the level of the control group at the end of week 20 (Figure 4B). In a study on the spinal dorsal column transection model in C3 of adult female Fischer rats, Jones et al. (2002) stated that NG2 was the most important upregulated proteoglycan among CSPGs family members after SCI.

The immunohistochemical results of Jones’ study reflected an upregulation of NG2, 24 hours after the injury, and this maximum level remained until about 7 weeks after the injury. However, an average upregulation was observed in proteoglycans brevican, versican, and neurocan, 7 days after the injury, and the phosphagen level downregulated, unlike other proteoglycans. These researchers stated that NG2 was more expressed in the caudal region of the injury than its rostral region; therefore, they introduced NG2 as the main component for inhibitory molecules in the ECM that probably inhibits axonal regeneration. The discrepancy between results reported by Tang and Jones and our findings may be due to differences in the methods, the type, the severity of injury, and even the animals species used.

These researchers used western blotting and immunohistochemical methods, dorsal column transection at C1–C2, or C3 segments of adult female rats of Sprague and Fischer rats. However, Andrews et al. (2012) used the western blotting and immunohistochemical techniques to measure the expression of aggrecan, neurocan, brevican, and NG2 proteoglycans in a severe contusion SCI model in the spinal segments far from the injury site (cervical and lumbar areas) of adult female Sprague rats 3,7,14, and 28 days after the injury. They observed an increase in neurocan level inside and outside of the injury location, while there was a sharp decline in aggrecan and brevican levels at the injury site and remained unchanged in the distal segments. These researchers also argued that the NG2 level remained unchanged in the injured site and cervical region and was increased in the lumbar region.

Some researchers believed that neurocan and NG2 levels have slightly increased in segments surrounding the injury in rodent compression models (Iaci, Vecchione, Zimber, & Caggiano, 2007). The GAG staining (Lemons, Howland, & Anderson, 1999) and immune-reactivity of NG2 (McTigue, Tripathi, & Wei, 2006) increased after the contusion injury. The upregulation of versican V2, aggrecan, and neurocan as CSPGs family members, following multiple sclerosis, was also reported by Sobel & Ahmed (2001). According to the present study data, which demonstrated a higher concentration of DS than CS, it seems that DS plays a major inhibitory role, compared to the CS, in the glial scar of Wistar rats with the contused spinal cord.

On the other hand, considering the high concentration of these proteoglycans, by the end of the second week after the injury, it seems that unsatisfactory success in therapeutic interventions by previous studies, in which interventions have been conducted early, may be due to the impermeability of glial scar caused by the high concentration of its inhibitors. However, to reach an exact conclusion, further studies are required with larger sample sizes and use of other animal species, and other complementary techniques, like western blotting and immunohistochemistry.

## 5. Conclusion

The histological results of this study suggested that the relative motor function recovery observed in the groups over time, though limited, can be partly due to axonal regeneration and remyelination. Reduced injury DS and CS levels observed by the ELISA technique also supported the permeability of glial scar at the end times of study. Therefore, the resulting motor functional recovery over time seems to be partly due to reduced levels of CS and DS inhibitory molecules, especially DS in the glial scar. Additionally, considering the high level of DS, compared with CS in the scar, as well as a reduction in its level from the second week onwards, the second week after the injury is the optimal time for the therapeutic intervention in terms of scar permeability and earlier interventions will yield no satisfactory results due to the high concentration of the abovementioned inhibitory factors.

## References

[B1] Andrews E. M. Richards R. J. Yin F. Q. Viapiano M. S. Jakeman L. B. (2012). Alterations in chondroitin sulfate proteoglycan expression occur both at and far from the site of spinal contusion injury. Experimental Neurology, 235(1), 174–87. [DOI:10.1016/j.expneurol.2011.09.008] [PMID ] [PMCID ] 21952042 PMC3640493

[B2] Basso D. M. Beattie M. S. Bresnahan J. C. (1996). Graded histological and locomotor outcomes after spinal cord contusion using the NYU weight-drop device versus transection. Experimental Neurology, 139(2), 244–56. [DOI:10.1006/exnr.1996.0098] [PMID ] 8654527

[B3] Bradford M. M. (1976). A rapid and sensitive method for the quantitation of microgram quantities of protein utilizing the principle of protein-dye binding. Analytical Biochemistry, 72(1–2), 248–54. [DOI:10.1016/0003-2697(76)90527-3] 942051

[B4] Devivo M. (2003). Epidemiology of spinal cord injury. New York: Demos Medical Publishing.

[B5] Faulkner J. R. Herrmann J. E. Woo M. J. Tansey K. E. Doan N.B. Sofroniew M. V. (2004). Reactive astrocytes protect tissue and preserve function after spinal cord injury. The Journal of Neuro-science, 24(9), 2143–55. [DOI:10.1523/JNEUROSCI.3547-03.2004] [PMID ] [PMCID ] PMC6730429 14999065

[B6] Fawcett J. W. Asher R. A. (1999). The glial scar and central nervous system repair. Brain Research Bulletin, 49(6), 377–91. [DOI:10.1016/S0361-9230(99)00072-6] 10483914

[B7] Fitch M. T. Silver J. (2008). CNS injury, glial scars, and inflammation: Inhibitory extracellular matrices and regeneration failure. Experimental Neurology, 209(2), 294–301. [DOI:10.1016/j.expneurol.2007.05.014] [PMID ] [PMCID ] 17617407 PMC2268907

[B8] Fouad K. Schnell L. Bunge M. B. Schwab M. E. Liebscher T. Pearse D. D. (2005). Combining Schwann cell bridges and olfactory-ensheathing glia grafts with chondroitinase promotes locomotor recovery after complete transection of the spinal cord. The Journal of Neuroscience, 25(5), 1169–78. [DOI:10.1523/JNEUROSCI.3562-04.2005] [PMID ] [PMCID ] 15689553 PMC6725952

[B9] Garcia-Alias G. Lopez-Vales R. Fores J. Navarro X. Verdu E. (2004). Acute transplantation of olfactory ensheathing cells or Schwann cells promotes recovery after spinal cord injury in the rat. Journal of Neuroscience Research, 75(5), 632–41. [DOI:10.1002/jnr.20029] [PMID ] 14991839

[B10] Iaci J. F. Vecchione A. M. Zimber M. P. Caggiano A. O. (2007). Chondroitin sulfate proteoglycans in spinal cord contusion injury and the effects of chondroitinase treatment. Journal of Neurotrauma, 24(11), 1743–59. [DOI:10.1089/neu.2007.0366] [PMID ] 18001203

[B11] Jones L. L. Margolis R. U. Tuszynski M. H. (2003). The chondroitin sulfate proteoglycans neurocan, brevican, phosphacan, and versican are differentially regulated following spinal cord injury. Experimental Neurology, 182(2), 399–411. [DOI:10.1016/S0014-4886(03)00087-6] 12895450

[B12] Jones L. L. Yamaguchi Y. Stallcup W. B. Tuszynski M. H. (2002). NG2 is a major chondroitin sulfate proteoglycan produced after spinal cord injury and is expressed by macrophages and oligodendrocyte progenitors. The Journal of Neuroscience, 22(7), 2792–803. [DOI:10.1523/JNEUROSCI.22-07-02792.2002] [PMID ] [PMCID ] 11923444 PMC6758310

[B13] Lau L. W. Cua R. Keough M. B. Haylock-Jacobs S. Yong V.W. (2013). Pathophysiology of the brain extracellular matrix: A new target for remyelination. Nature Reviews Neuroscience, 14(10), 722–9. [DOI:10.1038/nrn3550] [PMID ] 23985834

[B14] Lemons M. L. Howland D. R. Anderson D. K. (1999). Chondroitin sulfate proteoglycan immunoreactivity increases following spinal cord injury and transplantation. Experimental Neurology, 160(1), 51–65. [DOI:10.1006/exnr.1999.7184] [PMID ] 10630190

[B15] Massey J. M. Amps J. Viapiano M. S. Matthews R. T. Wagoner M. R. Whitaker CM (2008). Increased chondroitin sulfate proteoglycan expression in denervated brainstem targets following spinal cord injury creates a barrier to axonal regeneration overcome by chondroitinase ABC and neurotrophin-3. Experimental Neurology, 209(2), 426–45. [DOI:10.1016/j.expneurol.2007.03.029] [PMID ] [PMCID ] 17540369 PMC2270474

[B16] Matsui F. Oohira A. (2004). Proteoglycans and injury of the central nervous system. Congenital Anomalies, 44(4),181–8. [DOI:10.1111/j.1741-4520.2004.00038.x] [PMID ] 15566408

[B17] McTigue D. M. Tripathi R. Wei P. (2006). NG2 colocalizes with axons and is expressed by a mixed cell population in spinal cord lesions. Journal of Neuropathology and Experimental Neurology, 65(4), 406–20. [DOI:10.1097/01.jnen.0000218447.32320.52] [PMID ] 16691121

[B18] Mothe A. J. Tator C. H. (2005). Proliferation, migration, and differentiation of endogenous ependymal region stem/progenitor cells following minimal spinal cord injury in the adult rat. Neuro-science, 131(1), 177–87. [DOI:10.1016/j.neuroscience.2004.10.011] [PMID ] 15680701

[B19] Myer D. J. Gurkoff G. G. Lee S. M. Hovda D. A. Sofroniew M. V. (2006). Essential protective roles of reactive astrocytes in traumatic brain injury. Brain, 129(10), 2761–72. [DOI:10.1093/brain/awl165] [PMID ] 16825202

[B20] Norenberg M. D. Smith J. Marcillo A. (2004). The pathology of human spinal cord injury: Defining the problems. Journal of Neurotrauma, 21(4), 429–40. [DOI:10.1089/089771504323004575] [PMID ] 15115592

[B21] Okada S. Nakamura M. Katoh H. Miyao T. Shimazaki T. Ishii K. (2006). Conditional ablation of Stat3 or Socs3 discloses a dual role for reactive astrocytes after spinal cord injury. Nature Medicine, 12(7), 829–34. [DOI:10.1038/nm1425] [PMID ] 16783372

[B22] Pasterkamp R. J. Anderson P. N. Verhaagen J. (2001). Peripheral nerve injury fails to induce growth of lesioned ascending dorsal column axons into spinal cord scar tissue expressing the axon repellent Semaphorin3A. The European Journal of Neuroscience, 13(3), 457–71. [DOI:10.1046/j.0953-816X.2000.01398.x] [PMID ] 11168552

[B23] Plant G. W. Bates M. L. Bunge M. B. (2001). Inhibitory proteoglycan immunoreactivity is higher at the caudal than the rostrals schwann cell graft-transected spinal cord interface. Molecular and Cellular Neuroscience, 17(3), 471–87. [DOI:10.1006/mcne.2000.0948] [PMID ] 11273643

[B24] Raineteau O. Fouad K. Bareyre F. M. Schwab M. E. (2002). Reorganization of descending motor tracts in the rat spinal cord. The European journal of neuroscience, 16(9), 1761–71. [DOI:10.1046/j.1460-9568.2002.02243.x] [PMID ] 12431229

[B25] Rhodes K. E. Fawcett J. W. (2004). Chondroitin sulphate proteoglycans: Preventing plasticity or protecting the CNS? Journal of Anatomy, 204(1), 33–48. [DOI:10.1111/j.1469-7580.2004.00261.x] [PMID ] [PMCID ] 14690476 PMC1571240

[B26] Richter M. W. Fletcher P. A. Liu J. Tetzlaff W. Roskams A. J. (2005). Lamina propria and olfactory bulb ensheathing cells exhibit differential integration and migration and promote differential axon sprouting in the lesioned spinal cord. The Journal of Neuroscience, 25(46), 10700–11. [DOI:10.1523/JNEUROSCI.3632-05.2005] [PMID ] [PMCID ] 16291943 PMC6725847

[B27] Rolls A. Shechter R. Schwartz M. (2009). The bright side of the glial scar in CNS repair. Nature Reviews Neuroscience, 10(3), 235–41. [DOI:10.1038/nrn2591] [PMID ] 19229242

[B28] Ropper A. H. (2001). Traumatic injuries of the head and spine. New York: McGraw-Hill.

[B29] Shields L. B. Zhang Y. P. Burke D. A. Gray R. Shields C. B. (2008). Benefit of chondroitinase ABC on sensory axon regeneration in a laceration model of spinal cord injury in the rat. Surgical Neurology, 69(6), 568–77. [DOI:10.1016/j.surneu.2008.02.009] [PMID ] [PMCID ] 18486695 PMC2504084

[B30] Siebert J. R. Osterhout D. J. (2011). The inhibitory effects of chondroitin sulfate proteoglycans on oligodendrocytes. Journal of Neurochemistry, 119(1), 176–88. [DOI:10.1111/j.1471-4159.2011.07370.x] [PMID ] 21848846

[B31] Silver J. Miller J. H. (2004). Regeneration beyond the glial scar. Nature Reviews Neuroscience, 5(2), 146–56. [DOI:10.1038/nrn1326] [PMID ] 14735117

[B32] Sobel R. A. Ahmed A. S. (2001). White matter extracellular matrix chondroitin sulfate/dermatan sulfate proteoglycans in multiple sclerosis. Journal of Neuropathology and experimental Neurology, 60(12), 1198–207. [DOI:10.1093/jnen/60.12.1198] [PMID ] 11764092

[B33] Sofroniew M. V. (2009). Molecular dissection of reactive astrogliosis and glial scar formation. Trends in Neurosciences, 32(12), 638–47. [DOI:10.1016/j.tins.2009.08.002] [PMID ] [PMCID ] 19782411 PMC2787735

[B34] Tang X. Davies J. E. Davies S. J. (2003). Changes in distribution, cell associations, and protein expression levels of NG2, neurocan, phosphacan, brevican, versican V2, and tenascin-C during acute to chronic maturation of spinal cord scar tissue. Journal of Neuroscience Research, 71(3), 427–44. [DOI:10.1002/jnr.10523] [PMID ] 12526031

[B35] Ueno M. Yamashita T. (2008). Strategies for regenerating injured axons after spinal cord injury: Insights from brain development. Biologics: Targets & Therapy, 2(2), 253–64. [DOI:10.2147/BTT.S2715] [PMID ] [PMCID ] 19707358 PMC2721354

[B36] Verdu E. Garcia-Alias G. Fores J. Lopez-Vales R. Navarro X. (2003). Olfactory ensheathing cells transplanted in lesioned spinal cord prevent loss of spinal cord parenchyma and promote functional recovery. Glia, 42(3), 275–86. [DOI:10.1002/glia.10217] [PMID ] 12673833

[B37] Wang C. Y. Chen J. K. Wu Y. T. Tsai M. J. Shyue S. K. Yang C. S. (2011). Reduction in antioxidant enzyme expression and sustained inflammation enhance tissue damage in the subacute phase of spinal cord contusive injury. Journal of Biomedical Science, 18(1), 13. [DOI:10.1186/1423-0127-18-13] [PMID ] [PMCID ] 21299884 PMC3040708

[B38] Weidner N. Ner A. Salimi N. Tuszynski M. H. (2001). Spontaneous corticospinal axonal plasticity and functional recovery after adult central nervous system injury. Proceedings of the National Academy of Sciences of the United States of America, 98(6), 3513–8. [DOI:10.1073/pnas.051626798] [PMID ] [PMCID ] 11248109 PMC30684

[B39] Widenfalk J. Lundstromer K. Jubran M. Brene S. Olson L. (2001). Neurotrophic factors and receptors in the immature and adult spinal cord after mechanical injury or kainic acid. The Journal of Neuroscience, 21(10), 3457–75. [DOI:10.1523/JNEUROSCI.21-10-03457.2001] [PMID ] [PMCID ] 11331375 PMC6762497

[B40] Wilson J. R. Forgione N. Fehlings M. G. (2013). Emerging therapies for acute traumatic spinal cord injury. Canadian Medical Association Journal, 185(6),485–92. [DOI:10.1503/cmaj.121206] [PMID ] [PMCID ] 23228995 PMC3612151

